# Association of Marginalized Identities With Alpha Omega Alpha Honor Society and Gold Humanism Honor Society Membership Among Medical Students

**DOI:** 10.1001/jamanetworkopen.2022.29062

**Published:** 2022-09-07

**Authors:** Katherine A. Hill, Mayur M. Desai, Sarwat I. Chaudhry, Mytien Nguyen, William McDade, Yunshan Xu, Fangyong Li, Tonya Fancher, Alexandria M. Hajduk, Marjorie J. Westervelt, Dowin Boatright

**Affiliations:** 1 Yale School of Medicine, New Haven, Connecticut; 2 Department of Chronic Disease Epidemiology, Yale School of Public Health, New Haven, Connecticut; 3 Section of General Internal Medicine, Yale School of Medicine, New Haven, Connecticut; 4 Accreditation Council for Graduate Medical Education, Chicago, Illinois; 5 Yale Center for Analytic Sciences, Yale School of Public Health, New Haven, Connecticut; 6 Department of Internal Medicine and Office of Workforce Innovation and Community Engagement, University of California Davis; 7 Training Program in Geriatric Clinical Epidemiology & Aging-Related Research, Yale School of Medicine, New Haven, Connecticut; 8 Workforce Innovation and Community Engagement, University of California Davis; 9 Department of Emergency Medicine, New York University Grossman School of Medicine, New York

## Abstract

**Importance:**

Disparities in medical student membership in Alpha Omega Alpha (AOA) are well documented. Less is known about Gold Humanism Honor Society (GHHS) membership and it remains unknown how the intersection of different identities is associated with membership in these honor societies.

**Objective:**

To examine the association between honor society membership and medical student race and ethnicity, sex, sexual orientation, socioeconomic status, and intersection of identities.

**Design, Setting, and Participants:**

This cross-sectional study analyzed data from Association of American Medical Colleges data collection instruments. The study included all students who graduated from Liaison Committee on Medical Education–accredited US medical schools from 2016 to 2019 and completed the Graduation Questionnaire. Data analysis was conducted from January 12 to July 12, 2022.

**Main Outcomes and Measures:**

Likelihood of AOA and GHHS membership by student race and ethnicity, sex, sexual orientation, childhood family income, and intersection of identities.

**Results:**

The sample of 50 384 individuals comprised 82 (0.2%) American Indian or Alaska Native, 10 601 (21.0%) Asian, 2464 (4.9%) Black, 3291 (6.5%) Hispanic, 25 (0.1%) Native Hawaiian or Pacific Islander, 30 610 (60.8%) White, 2476 (4.9%) multiracial students, and 834 (1.7%) students of other races or ethnicities. Sex and sexual orientation included 25 672 (51.0%) men and 3078 (6.1%) lesbian, gay, and bisexual (LGB). Childhood family income comprised 31 758 (60.0%) individuals with $75 000 per year or greater, 8160 (16.2%) with $50 000 to $74 999 per year, 6864 (13.6%) with $25 000 to $49 999 per year, and 3612 (7.2%) with less than $25 000 per year. The sample included 7303 (14.5%) AOA members only, 4925 (9.8%) GHHS members only, and 2384 (4.7%) members of both societies. In AOA, American Indian or Alaska Native (OR, 0.49; 95% CI, 0.25-0.96), Asian (OR, 0.49; 95% CI, 0.45-0.53), Black (OR, 0.25; 95% CI, 0.20-0.30), Hispanic (OR, 0.53; 95% CI, 0.47-0.59), multiracial (OR, 0.69; 95% CI, 0.62-0.77), and other race and ethnicity (OR, 0.73; 95% CI, 0.60-0.88) were underrepresented compared with White students; LGB students (OR, 0.75; 95% CI, 0.67-0.83) were underrepresented compared with heterosexual students; and childhood family income $50 000 to $74 999 (OR, 0.81; 95% CI, 0.75-0.86), $25 000 to $49 999 (OR, 0.68; 95% CI, 0.62-0.74), and less than $25 000 (OR, 0.60; 95% CI, 0.53-0.69) were underrepresented compared with greater than or equal to $75 000. In GHHS, Asian students (OR, 0.80; 95% CI, 0.73-0.87) were underrepresented compared with White students, female students (OR, 1.55; 95% CI, 1.45-1.65) were overrepresented compared with male students, LGB students (OR, 1.36; 95% CI, 1.23-1.51) were overrepresented compared with heterosexual students, and students with childhood family income $25 000 to $49 999 (OR, 0.85; 95% CI, 0.78-0.94) and less than $25 000 (OR, 0.75; 95% CI, 0.66-0.86) were underrepresented compared with those with greater than or equal to $75 000. Likelihood of AOA, but not GHHS, membership decreased as number of marginalized identities increased.

**Conclusions and Relevance:**

In this cross-sectional study of US medical students, membership disparities were noted in both AOA and GHHS. However, differences in GHHS existed across fewer identities, sometimes favored the marginalized group, and were not cumulative.

## Key Points

**Question:**

Are there disparities in Alpha Omega Alpha (AOA) and Gold Humanism Honor Society (GHHS) membership based on student race and ethnicity, sex, sexual orientation, socioeconomic status, and intersection of different identities?

**Findings:**

In this cross-sectional study of 50 384 medical students, membership disparities were found in both honor societies, but disparities in AOA existed across more identities, were often larger, and increased with increasing numbers of marginalized identities. Disparities in GHHS membership sometimes favored the marginalized group and no cumulative disadvantage was seen for students with multiple marginalized identities.

**Meaning:**

The findings of this study suggest marginalized students are better represented in GHHS than in AOA.

## Teaser

This cross-sectional study examines the characteristics of students in medical school honor societies.

## Introduction

Alpha Omega Alpha (AOA) and Gold Humanism Honor Society (GHHS) are exclusive medical student honor societies.^1^ Membership can serve as an important steppingstone for advancement in medicine.^1,2,3,4,5,6^ Alpha Omega Alpha, founded in 1902 to promote “personal honesty and the spirit of medical research,” counts 55 Nobel Prize laureates and over 75% of medical school deans among its members.^7^ Gold Humanism Honor Society, founded in 2002 to promote humanism in medicine, has chapters at 137 US Liaison Committee on Medical Education–accredited medical schools and more than 40 000 members.^8,9^

Medical students reap benefits from honor society membership, including scholarships,^10^ mentorship,^11^ and networking^11^ opportunities. Individuals are asked to report AOA and GHHS membership status on the application for graduate medical education.^12^ Those who are AOA and GHHS members are more likely to obtain interviews^1,3^ for and match into graduate medical education^6^ than nonmembers. Even after residency, honor society membership is associated with success in academic medicine, including higher faculty rank and more citations of one’s published work.^2^

Honor society membership could be especially important for students from minoritized groups who often lack robust mentorship^13,14,15,16^ and frequently report being denied opportunities based on aspects of their identities.^17^ Prior research reported Asian, Black, and Hispanic students, as well as those of low socioeconomic status (SES), are less likely than their peers to be members of AOA, even after controlling for academic performance.^18,19,20^ Less is known about GHHS. One study noted that women were more likely than men to be GHHS members, and no major difference existed between students who were Black or White.^20^ However, this study did not investigate differences by races and ethnicities other than Black and White or identities such as SES or sexual orientation.

Moreover, little is known about the intersection between different identities and honor society membership. To address these critical gaps, we examined AOA and GHHS membership in a national sample of medical students. To our knowledge, this study is the largest to examine both AOA and GHHS membership and the first to examine honor society membership using an intersectional analysis.

## Methods

### Study Design and Population

We conducted a retrospective cross-sectional study using deidentified data from the Association of American Medical Colleges American Medical College Application Service,^21^ Electronic Residency Application Service (ERAS),^12^ and graduation questionnaire.^22^ Our cohort included all US medical students who graduated from Liaison Committee on Medical Education–accredited schools from 2016 to 2019, and completed the graduation questionnaire. Data analysis was conducted from January 12 to July 12, 2021. This study was approved by the Yale University Institutional Review Board and followed the Strengthening the Reporting of Observational Studies in Epidemiology (STROBE) reporting guideline for cross-sectional studies.

### Variables

Dependent variables included student-reported AOA and GHHS membership status via ERAS. Independent variables included, sex assigned at birth (male vs female) and self-reported race and ethnicity, sexual orientation (heterosexual vs lesbian, gay or bisexual [LGB]), and childhood SES based on income (<$25 000, $25 000-$49 999, $50 000-$74 999, and≥$75 000). The Medical College Admission Test (MCAT) score quartile was included as a potential mediating variable.^18,19^ All demographic variables and the MCAT score were obtained from the American Medical Colleges data collection instruments.

For nonintersectional analyses, race and ethnicity categorization followed student self-report as American Indian or Alaska Native; Asian; Black or African American; Hispanic, Latino, or of Spanish origin; Native Hawaiian or Pacific Islander, non-Hispanic White, or other. Students who selected White and Hispanic were categorized as Hispanic. Students who selected any other combination of multiple races and ethnicities were categorized as multiracial.

For intersectional analyses, we dichotomized both race and ethnicity and childhood family income. Because of research noting Asian students and students from races and ethnicities underrepresented in medicine are less likely to be AOA members,^18,19^ we dichotomized race and ethnicity as non-Hispanic White vs non-White. We dichotomized childhood family income as low income vs non–low income. The low-income cutoff was $50 000, or approximately 200% of the federal poverty level for a family of 4 averaged from 2016 to 2019.^23^

Our initial sample included 63 720 students. Students were excluded from all analyses if they attended a historically Black college or university, a Puerto Rican medical school, or a school that did not have graduates who participated in each of the 4 years (2016-2019) of GQ survey records used (2442 [3.8%]). Students were also excluded if their school did not have a GHHS chapter (4858 [7.6%]) or if their school did not have an AOA chapter or if AOA membership data were missing from ERAS because their school chose members during their senior year (8880 [13.9%]). The final sample included 50 384 students; of these, 159 (0.3%) were missing data for race and ethnicity, 2 were missing sex (<0.1%), 3955 were missing sexual orientation (7.8%), 13 266 (26.3%) were missing childhood family income, and 1759 (3.5%) were missing MCAT scores.

### Statistical Analysis

Multiple imputation was performed using a fully conditional specification method, which uses a separate conditional distribution of each imputed variable and allows for arbitrary missing patterns, to impute all missing data. Specifically, categorical missing values were imputed using fully conditional specification discriminant function, and continuous variables were imputed using fully conditional specification regression function. We included all individual sociodemographic variables, institutional variables, and membership outcome variables in the imputation model. Twenty imputed data sets were created using SAS procedure PROC MI and analyzed separately. The results of all data sets were summarized using PROC MIANALYZE.

*P* values were found, using 2-sided, unpaired testing, by combining χ^2^ statistics from 20 imputed data sets. For nonintersectional analyses, we used logistic regression to model the association between demographic variables and honor society membership, adjusting for clustering by school, MCAT score, and demographic variables. For intersectional analyses, we examined 16 unique possible combinations using 4 demographic variables (sex, race and ethnicity, sexual orientation, and childhood family income). We performed logistic regression to model the association between all 16 demographic combinations and honor society membership, adjusting for clustering by school and MCAT score. Because sample characteristics (eTable in the Supplement) and results were similar with and without imputation, only results from multiple imputation are presented. Statistical analyses were performed using SAS, version 9.4 (SAS Institute Inc) and *P* < .05 was set as the threshold for statistical significance.

## Results

The final sample of 50 384 students (Table 1) included 82 (0.2%) American Indian or Alaska Native, 10 601 (21.0%) Asian, 2464 (4.9%) Black, 3291 (6.5%) Hispanic, 25 (0.1%) Native Hawaiian or Pacific Islander, 30 610 (60.8%) White, and 2476 (4.9%) multiracial students. A total of 834 students (1.7%) listed their race and ethnicity as other. In our sample, 24 712 students (49.0%) were women, 25 672 (51.0%) were men, and 3078 (6.1%) were LGB. Childhood family income was greater than or equal to $75 000 for 31 758 students (60.0%), $50 000 to $74 999 for 8160 (16.2%), $25 000 to $49 999 for 6864 (13.6%), and less than $25 000 for 3612 students (7.2%). Our sample included 7303 students (14.5%) who were AOA members only, 4925 (9.8%) GHHS members only, and 2384 (4.7%) members of both societies.

**Table 1.** Sample Demographic Characteristics

Characteristic	AOA member			GHHS member		
	No	Yes	*P* value	No	Yes	*P* value
No. (%)	40 697 (80.8)	9687 (19.2)		43 075 (85.5)	7309 (14.5)	
Race						
American Indian or Alaska Native	75 (91.5)	7 (8.5)	<.001	72 (87.8)	10 (12.2)	<.001
Asian	9148 (86.3)	1453 (13.7)		9313 (87.8)	1288 (12.2)	
Black or African American	2355 (95.6)	108 (4.4)		2052 (83.3)	412 (16.7)	
Hispanic, Latino, or of Spanish origin	2937 (89.2)	355 (10.8)		2806 (85.3)	485 (14.7)	
Multiracial	2069 (83.6)	407 (16.4)		2095 (84.6)	381 (15.4)	
Native Hawaiian or Pacific Islander	23 (88.5)	3 (11.5)		24 (92.3)	2 (7.7)	
White	23 397 (76.4)	7213 (23.6)		26 013 (85.0)	4597 (15.0)	
Other	693 (83.1)	141 (16.9)		700 (83.9)	134 (16.1)	
Sex						
Male	20 747 (80.8)	4925 (19.2)	>.99	22 633 (88.2)	3039 (11.8)	<.001
Female	19 950 (80.7)	4762 (19.3)		20 442 (82.7)	4270 (17.3)	
Sexual orientation						
Heterosexual	38 111 (80.6)	9196 (19.4)	.38	40 552 (85.7)	6754 (14.3)	.04
LGB	2586 (84.0)	491 (16.0)		2523 (82.0)	555 (18.0)	
Childhood family income, $						
≥75 000	24 681 (77.7)	7067 (22.3)	<.001	26 946 (84.9)	4802 (15.1)	.10
50 000-74 999	6778 (83.1)	1382 (16.9)		7005 (85.8)	1156 (14.2)	
25 000-49 999	5973 (87.0)	891 (13.0)		5952 (86.7)	912 (13.3)	
<25 000	3265 (90.4)	347 (9.6)		3172 (87.8)	439 (12.2)	
MCAT score						
<29	9260 (89.9)	1044 (10.1)	<.001	8663 (84.1)	1642 (15.9)	.003
29-31	12 500 (82.2)	2715 (17.8)		12 972 (85.3)	2243 (14.7)	
32-33	8192 (77.5)	2382 (22.5)		9021 (85.3)	1553 (14.7)	
≥34	10 745 (75.2)	3546 (24.8)		12 419 (86.9)	1871 (13.1)	

Abbreviations: AOA, Alpha Omega Alpha; GHHS, Gold Humanism Honor Society; LGB, lesbian, gay, and bisexual.

### AOA Membership

Results showed 7213 (23.6%) White students were AOA members, compared with 7 (8.5%) American Indian or Alaska Native, 1453 (13.7%) Asian, 108 (4.4%) Black, 355 (10.8%) Hispanic, 407 (16.4%) multiracial, and 141 (16.9%) other race and ethnicity students (*P* < .001) (Table 1). No statistically significant difference was found in AOA membership between male (4925 [19.2%]) and female (4762 [19.3%]) (*P* > .99) or heterosexual (9196 [19.4%]) and LGB (491 [16.0%]) (*P* = .38) students. A higher percentage of students with childhood family income greater than or equal to $75 000 was AOA members (7067 [22.3%]) than were students with childhood family incomes of $50 000 to $74 999 (1382 [16.9%]), $25 000 to $49 999 (891 [13.0%]), and less than $25 000 (347 [9.6%]) (*P* < .001).

After adjusting for clustering by medical school, all demographic variables, and MCAT score, American Indian or Alaska Native (odds ratio [OR] 0.49; 95% CI, 0.25- 0.96), Asian (OR, 0.49; 95% CI, 0.45-0.53), Black (OR, 0.25; 95% CI, 0.20-0.30), Hispanic (OR, 0.53; 95% CI, 0.47-0.59), multiracial (OR, 0.69; 95% CI, 0.62-0.77) students, and those of other race and ethnicity (OR, 0.73; 95% CI, 0.60-0.88) were less likely to be AOA members than were White students (Table 2). Female students were more likely than male students (OR, 1.16; 95% CI, 1.10-1.23) and LGB were less likely than heterosexual students (OR, 0.79; 95% CI, 0.71-0.87) to be AOA members. Students with childhood family incomes of $50 000 to $74 999 (OR, 0.81; 95% CI, 0.75-0.86), $25 000 to $49 999 (OR, 0.68; 95% CI, 0.62-0.74), and less than $25 000 (OR, 0.60; 95% CI, 0.53-0.69) had lower odds of AOA membership than did students with childhood family incomes greater than or equal to $75 000. There was also an association between MCAT score and AOA membership (29-31: OR, 1.66; 95% CI, 1.50-1.83; 32-33: OR, 2.25; 95% CI, 1.99-2.54; ≥34: OR, 2.60; 95% CI, 2.27-2.97) (Table 2).

**Table 2.** Odds of Alpha Omega Alpha Membership

Characteristic	Adjusted odds ratio (95% CI)		
	Model 1^a^	Model 2^b^	Model 3^c^
Race			
American Indian or Alaska Native	0.30 (0.15-0.60)	0.35 (0.17-0.70)	0.49 (0.25-0.96)
Asian	0.52 (0.48-0.56)	0.55 (0.50-0.59)	0.49 (0.45-0.53)
Black or African American	0.15 (0.12-0.18)	0.18 (0.15-0.22)	0.25 (0.20-0.30)
Hispanic, Latino, or of Spanish origin	0.39 (0.34-0.45)	0.45 (0.40-0.51)	0.53 (0.47-0.59)
Multiracial	0.64 (0.57-0.72)	0.68 (0.60-0.76)	0.69 (0.62-0.77)
Native Hawaiian or other Pacific Islander	0.42 (0.13-1.36)	0.49 (0.15-1.61)	0.55 (0.17-1.76)
White	1 [Reference]	1 [Reference]	1 [Reference]
Other	0.66 (0.55-0.80)	0.74 (0.62-0.90)	0.73 (0.60-0.88)
Sex			
Male	1 [Reference]	1 [Reference]	1 [Reference]
Female	1.01 (0.95-1.07)	1.05 (0.99-1.12)	1.16 (1.10-1.23)
Sexual orientation			
Heterosexual	1 [Reference]	1 [Reference]	1 [Reference]
LGB	0.79 (0.71-0.87)	0.78 (0.71-0.87)	0.75 (0.67-0.83)
Childhood family income			
≥$75 000	1 [Reference]	1 [Reference]	1 [Reference]
$50 000-$74 999	0.71 (0.66-0.76)	0.76 (0.71-0.81)	0.81 (0.75-0.86)
$25 000-$49 999	0.52 (0.48-0.57)	0.62 (0.57-0.68)	0.68 (0.62-0.74)
<$25 000	0.37 (0.33-0.42)	0.53 (0.46-0.60)	0.60 (0.53-0.69)
MCAT score			
<29	NA	NA	1 [Reference]
29-31	NA	NA	1.66 (1.50-1.83)
32-33	NA	NA	2.25 (1.99-2.54)
≥34	NA	NA	2.60 (2.27-2.97)

Abbreviations: LGB, lesbian, gay, and bisexual; MCAT, Medical College Admission Test; NA, not applicable.

^a^ Adjusted for clustering by school.

^b^ Adjusted for clustering by school and for demographic variables (race and ethnicity, sex, sexual orientation, and childhood family income).

^c^ Adjusted for clustering by school and for demographic variables and MCAT quartile.

As the number of marginalized identities that a student reported increased, the student’s odds of AOA membership decreased (Table 3). Only 5.6% of students with 4 marginalized identities (non-White, female, LGB, low- income: OR, 0.24; 95% CI, 0.11-0.50) were AOA members compared with 23.7% of students with no marginalized identities (White, male, heterosexual, non–low-income). White, female, heterosexual, non–low-income students were most likely to be AOA members, followed by students with no marginalized identities.

**Table 3.** Intersectional Analysis of AOA Membership

Unique identity group^a^	AOA Member		Adjusted odds ratio (95% CI)
	No	Yes	
White, male, heterosexual, non–low-income	10 020 (76.3)	3114 (23.7)	1 [Reference]
White, female, heterosexual, non–low-income	8665 (73.7)	3092 (26.3)	1.28 (1.20-1.37)
Non-White, male, heterosexual, non–low-income	4962 (84.7)	895 (15.3)	0.57 (0.51-0.63)
White, male, LGB, non–low-income	699 (78.8)	188 (21.2)	0.83 (0.71-0.99)
White, male, heterosexual, low-income	1852 (84.0)	353 (16.0)	0.67 (0.58-0.77)
Non-White, female, heterosexual, non–low-income	5869 (86.3)	932 (13.7)	0.56 (0.50-0.62)
White, female, LGB, non–low-income	586 (81.0)	138 (19.0)	0.78 (0.63-0.97)
White, female, heterosexual, low-income	1308 (82.1)	285 (17.9)	0.85 (0.73-0.99)
Non-White, male, LGB, non–low-income	352 (86.2)	56 (13.8)	0.51 (0.37-0.69)
Non-White, male, heterosexual, low-income	2474 (90.1)	271 (9.9)	0.42 (0.35-0.50)
White, male, LGB, low-income	152 (87.7)	21 (12.3)	0.49 (0.27-0.90)
Non-White, female, LGB, non–low-income	307 (90.2)	33 (9.8)	0.36 (0.24-0.53)
Non-White, female, heterosexual, low-income	2961 (92.1)	253 (7.9)	0.36 (0.31-0.42)
White, female, LGB, low-income	116 (84.3)	22 (15.7)	0.64 (0.39-1.05)
Non-White, male, LGB, low-income	237 (90.5)	25 (9.5)	0.39 (0.25-0.61)
Non-White, female, LGB, low-income	139 (94.4)	8 (5.6)	0.24 (0.11-0.50)

Abbreviations: AOA, Alpha Omega Alpha; LGB, lesbian, gay, and bisexual.

^a^ The non–low-income threshold was $50 000, or approximately 200% of the federal poverty level for a family of 4 averaged from 2016 to 2019. Non-White includes all students with self-reported race and ethnicity other than non-Hispanic White.

### GHHS Membership

Results showed that 4597 (15.0%) White students were GHHS members, compared with 10 (12.2%) American Indian or Alaska Native, 1288 (12.2%) Asian, 412 (16.7%) Black, 485 (14.7%) Hispanic, and 381 (15.4%) multiracial students, as well as 134 (16.1%) students of other race and ethnicity. A higher percentage of female (4270 [17.3%]) than male (3039 [11.8%]) (*P* < .001) students, and LGB (555 [18.0%]) than heterosexual students (6754 [14.3%]) (*P* = .04) were GHHS members (Table 1). No statistically significant differences in GHHS membership were found between students with childhood family income greater than or equal to $75 000 (4802 [15.1%]), $50 000 to $74 999 (1156 [14.2%]), $25 000 to $49 999 (912 [13.3%]), and less than $25 000 (439 [12.2%]) (*P* = .10).

After adjusting for clustering by medical school, all demographic variables, and MCAT score, Asian medical students (OR, 0.80; 95% CI, 0.73-0.87) were less likely than White students to be GHHS members. No statistically significant differences were found between White students and students of any other race and ethnicity. Female students were more likely than male students (OR, 1.55; 95% CI, 1.45-1.65) and LGB students were more likely than heterosexual (OR, 1.36; 95% CI, 1.23-1.51) to be GHHS members (Table 4). Students with childhood family incomes of $25 000 to $49 999 (OR, 0.85; 95% CI, 0.78-0.94) and less than $25 000 (OR, 0.75; 95% CI, 0.66-0.86) were less likely to be GHHS members than were students with childhood family incomes greater than or equal to $75 000. Students in the highest MCAT quartile had slightly higher odds of GHHS membership than did students in the lowest MCAT quartile (OR, 0.88; 95% CI, 0.78- 0.98; *P* = .02).

**Table 4.** Odds of Gold Humanism Honor Society Membership

Characteristic	Adjusted odds ratio (95% CI)		
	Model 1^a^	Model 2^b^	Model 3^c^
Race			
American Indian or Alaska Native	0.79 (0.44-1.40)	0.82 (0.46-1.44)	0.79 (0.45-1.39)
Asian	0.78 (0.72-0.85)	0.79 (0.72-0.85)	0.80 (0.73-0.87)
Black or African American	1.14 (1.00-1.29)	1.14 (1.00-1.30)	1.11 (0.96-1.27)
Hispanic, Latino, or of Spanish Origin	0.98 (0.87-1.10)	1.01 (0.89-1.15)	1.00 (0.88-1.13)
Multiracial	1.03 (0.93-1.14)	1.02 (0.92-1.14)	1.02 (0.92-1.13)
Native Hawaiian or other Pacific Islander	0.48 (0.11-2.15)	0.48 (0.10-2.20)	0.48 (0.10-2.19)
White	1 [Reference]	1 [Reference]	1 [Reference]
Other	1.09 (0.89-1.33)	1.15 (0.93-1.41)	1.15 (0.93-1.41)
Sex			
Male	1 [Reference]	1 [Reference]	1 [Reference]
Female	1.56 (1.46-1.66)	1.57 (1.47-1.67)	1.55 (1.45-1.65)
Sexual orientation			
Heterosexual	1 [Reference]	1 [Reference]	1 [Reference]
LGB	1.32 (1.19-1.46)	1.35 (1.22-1.50)	1.36 (1.23-1.51)
Childhood family income, $			
≥$75 000	1 [Reference]	1 [Reference]	1 [Reference]
50 000-74 999	0.93 (0.85-1.00)	0.93 (0.86-1.01)	0.92 (0.85-1.00)
25 000-49 999	0.86 (0.78-0.94)	0.86 (0.79-0.95)	0.85 (0.78-0.94)
<25 000	0.78 (0.68-0.88)	0.77 (0.67-0.87)	0.75 (0.66-0.86)
MCAT score			
<29	NA	NA	1 [Reference]
29-31	NA	NA	0.94 (0.86-1.03)
32-33	NA	NA	0.96 (0.87-1.07)
≥34	NA	NA	0.88 (0.78-0.98)

Abbreviations: LGB, lesbian, gay, and bisexual; NA, not applicable.

^a^ Adjusted for clustering by school.

^b^ Adjusted for clustering by school and for demographic variables (race and ethnicity, sex, sexual orientation, and socioeconomic status).

^c^ Ajusted for clustering by school and for demographic variables and MCAT quartile.

No clear association was seen between the number of marginalized identities and odds of GHHS membership (Table 5). Students with no marginalized identities were less likely to be GHHS members than students with 2 through 4 marginalized identities. However, likelihood of GHHS membership did not increase or decrease linearly with number of marginalized identities. Students with no marginalized identities were less likely to be members of GHHS than students from 8 of the unique identity groups. No significant difference was found between students with no marginalized identities and the 7 remaining groups.

**Table 5.** Intersectional Analysis of GHHS Membership

Unique identity group^a^	GHHS Member		Adjusted odds ratio (95% CI)
	No	Yes	
White, male, heterosexual, non–low-income	11542 (87.9)	1593 (12.1)	1 [Reference]
White, female, heterosexual, non–low-income	9598 (81.6)	2160 (18.4)	1.60 (1.47-1.74)
Non-White, male, heterosexual, non–low-income	5220 (89.1)	638 (10.9)	0.89 (0.79-1.00)
White, male, LGB, non–low-income	748 (84.4)	138 (15.6)	1.34 (1.10-1.63)
White, male, heterosexual, low-income	1960 (88.9)	244 (11.1)	0.89 (0.75-1.06)
Non-White, female, heterosexual, non–low-income	5667 (83.3)	1134 (16.7)	1.42 (1.28-1.58)
White, female, LGB, non–low-income	571 (78.9)	153 (21.1)	1.93 (1.56-2.38)
White, female, heterosexual, low-income	1342 (84.3)	251 (15.7)	1.31 (1.12-1.53)
Non-White, male, LGB, non–low-income	338 (82.8)	70 (17.2)	1.51 (1.16-1.96)
Non-White, male, heterosexual, low-income	2449 (89.2)	296 (10.8)	0.85 (0.72-1.01)
White, male, LGB, low-income	150 (86.6)	23 (13.4)	1.10 (0.66-1.82)
Non-White, female, LGB, non–low-income	268 (78.7)	72 (21.3)	1.94 (1.44-2.62)
Non-White, female, heterosexual, low-income	2774 (86.3)	439 (13.7)	1.09 (0.95-1.26)
White, female, LGB, low-income	103 (74.5)	35 (25.5)	2.45 (1.61-3.73)
Non-White, male, LGB, low-income	226 (86.2)	36 (13.8)	1.12 (0.75-1.67)
Non-White, female, LGB, low-income	120 (81.6)	27 (18.4)	1.55 (0.97-2.48)

Abbreviations: GHHS, Gold Humanism Honor Society; LGB, lesbian, gay, and bisexual.

^a^ The non–low-income threshold was $50 000, or approximately 200% of the federal poverty level for a family of 4 averaged from 2016 to 2019. Non-White includes all students with self-reported race and ethnicity other than non-Hispanic White.

## Discussion

We found that although there was underrepresentation of all racial and ethnic minoritized groups in AOA, this discrepancy did not hold true in GHHS. Within AOA, White students were 4 times more likely than Black students to be honor society members, students with childhood family income greater than or equal to $75 000 were almost twice as likely as students with childhood family income less than $25 000 to be members, and heterosexual students were 25% more likely than LGB students to be AOA members. These disparities persisted after adjustment for MCAT scores, a standardized examination considered by many medical schools as a marker of academic aptitude.

In contrast, within GHHS, there was parity in the representation of Black, Hispanic, and multiracial students compared with White students. Students who were LGB were more prevalent than heterosexual students in GHHS. In the fully adjusted model, female students were more likely than male students to be members of both AOA and GHHS. These findings are consistent with literature describing the representation of female compared with male students and Black compared with White students in GHHS^20^ and with previously described disparities in AOA membership by race and ethnicity and SES.^18,19^

Another key finding from our study is underrepresentation of Asian students and students from lower-income backgrounds in both AOA and GHHS. Although these findings warrant additional examination, they are consistent with previous research showing that individuals reporting Asian race are well represented in medicine and other high-paying fields but underrepresented in leadership positions.^24^ It is also consistent with research showing that nearly 15% of Asian medical students report experiencing discrimination in medical school and 4.4% of Asian medical students report being denied opportunities based on their race and ethnicity.^17^

Students with low SES may face several challenges in medical education that could influence honor society selection. Low-income students may need to engage in paid work to support themselves during medical school,^25,26^ leaving less time for extracurricular activities valued during the honor society selection process.^26^ Students from low SES backgrounds may face unique social and cultural obstacles,^25^ such as family-related stressors,^27^ lack of cultural capital^25^ and mentors,^25,26^ and difficulty navigating the often-opaque rules and structures governing academia.^25^

We found that women and LGB students were overrepresented in GHHS. It is possible the GHHS selection process is biased toward these groups; underlying causes of this finding merit investigation. Some studies have found female medical students are stereotyped as being more caring and compassionate than their male peers.^28,29^

It is also possible that women and LGB students are overrepresented because they are more likely to pursue activities valued by GHHS. Research suggests women are more likely than men to engage in community service,^30^ and female physicians are more likely than males to use patient-centered communication.^31^ In qualitative studies, lesbian, gay, bisexual, and transgender (LGBT) physicians and trainees report significant involvement in research, services, and programs that support LGBT health.^32,33^ Although not overrepresented in GHHS, students underrepresented in medicine by race and ethnicity may also have higher involvement in community service than their peers.^30^ Physicians and scientists underrepresented in medicine by race and ethnicity are more likely than their peers to serve underserved populations^34,35,36^ and to research critical subjects, including health disparities and patient-focused interventions.^37^

Another important finding from our study is the presence of cumulative disadvantage based on the number of a student’s marginalized identities in AOA membership. Students with no marginalized identities (White, male, heterosexual, non–low-income) were more than 4 times more likely to be AOA members than were students with 4 marginalized identities (non-White, female, LGB, low income). The finding of cumulative disadvantage is consistent with previous research showing marginalized identities can have an additive effect on academic^38^ and career^39^ success. In contrast, no cumulative disadvantage was seen in GHHS membership.

The distinct processes for member selection used by AOA and GHHS may contribute to member diversity or lack thereof. Historically, AOA used academic rank as a cutoff for membership eligibiligy.^40^ Academic rank is calculated differently among medical schools but often includes clerkship grades and standardized test scores.^41,42^ Predefined academic cutoffs may promote bias in deliberation and limit honor society membership diversity. Studies have shown racial and ethnic disparities in standardized test scores^43,44,45^ and clinical evaluations.^28,46,47,48^ Although this study controlled for standardized test scores via MCAT score, clinical evaluations may partially explain the disparities seen in AOA membership. Academic cutoffs may contribute to an amplification cascade that magnifies smaller preexisting biases against students who are historically marginalized in medicine.^47^

In contrast with AOA, GHHS uses student peer nomination to identify potential candidates.^49^ In peer nomination, medical students independently and anonymously identify classmates they believe best embody the values of GHHS.^49,50^ The efficacy of peer nomination is supported by numerous studies noting that the aggregated judgment of many independent individuals is more accurate than that of experts in isolation.^51,52^ Peer nomination accuracy and reliability have also been supported by research showing peer evaluations are consistent across raters^50,53,54,55^ and stable over time.^56^ Some research suggests peer evaluation may be a particularly useful assessment of interpersonal skills^50,57^ and may outperform both medical school grades and faculty evaluations as a factor estimating performance level during internship.^58^

After eligible candidates are identified, both societies use a deliberative body to finalize membership.^59^ These deliberative bodies may be another source of bias that could partially explain disparities in Asian and low SES student membership seen in both societies. Research suggests deliberative groups may show amplification of bias beyond that of individual members.^60,61^ While increasing the diversity of deliberative body members has been suggested as a possible solution to limit bias, studies have noted that individuals from historically marginalized groups, such as women and racial and ethnic minorities, may be reluctant to share unique information in these settings.^51,62,63^ Moreover, when individuals from marginalized groups share information, members of other groups may be less likely to listen to them compared with individuals perceived to have higher status.^51^

This study may have implications for the leaders of medical student honor societies, ERAS, residency programs, and medical schools. The GHHS peer nomination process deserves greater attention and could be a successful model to promote diversity, equity, and inclusion in honor society membership and in the receipt of academic awards in general. Nevertheless, both GHHS and AOA should consider examining how their current selection processes could be disadvantageous for Asian and low-income medical students.

Both AOA and GHHS may consider taking evidence-based steps to minimize bias by deliberative bodies. Encouraging committee members to share contrary decisions, anonymizing committee members’ votes, and assigning specific roles to committee members, for example, by making each member responsible for evaluating a different membership criterion, all have the potential to increase the participation of low-status committee members and reduce the social pressure to agree with higher-status members.^51^

One potential strategy to minimize bias in deliberative bodies is use of the Delphi technique.^51^ Although different variations of the Delphi technique exist, most involve an iterative process in which committee members provide feedback to each other using anonymous surveys. In the final step, committee members vote anonymously, using information gleaned during previous iterations to inform their selections.^51,64^ The Delphi technique ensures all committee members are able to contribute information equally to the deliberation and, because of its anonymous nature, limits social pressure to conform to the group consensus.^51^

The administrators of the ERAS application to graduate medical education training could examine the implications of including AOA membership status as a structured component of the ERAS application, given the well-documented and persistent disparities in membership by race and ethnicity, SES, and sexual orientation.^18,19,20^ When selecting trainees, residency program directors could consider honor society membership as part of a holistic process, recognizing the sociodemographic disparities possible in honor society membership. Because GHHS appears less likely than AOA to propagate structural barriers for students historically marginalized in medicine, residency program directors looking to promote equity and inclusion in their matriculant selection process may consider weighing GHHS membership more heavily than AOA membership.

In addition, following the example of University of California San Francisco,^65^ Mount Sinai,^66^ and Washington University at St Louis,^41^ among others, medical schools may consider conducting internal reviews of honor society membership for sociodemographic disparities. These reviews may incorporate a comprehensive view of diversity that includes race and ethnicity, sexual orientation, and SES, among other factors. If disparities are found, schools may consider identifying and dismantling barriers to honor society membership equity and inclusion.

### Limitations

This study has limitations. Our cohort had small numbers of Native Hawaiian or Pacific Islander students, likely contributing to the absence of statistically significant results for this group. Our study focused on sex, race and ethnicity, SES, and sexual orientation. However, those with other marginalized identities, such as individuals with disabilities and transgender or nonbinary students, may also face discrimination. Future studies should examine the representation of these and other groups in medical student honor societies. In addition, groups such as LGB and non-White may have included students from many different backgrounds that influenced their individual experience in medical school.

## Conclusions

In this cross-sectional study of US medical students, membership disparities were noted in both AOA and GHHS. However, differences in GHHS existed across fewer identities, sometimes favored the marginalized group, and were not cumulative. The findings of this study suggest marginalized students are better represented in GHHS than in AOA.

## Notes

Supplement. **eTable.** Sample Demographics With and Without Multiple Imputation
