# Supplementary material for: Association of Marginalized Identities With Alpha Omega Alpha Honor Society and Gold Humanism Honor Society Membership Among Medical Students
Source: JAMA Netw Open. 2022 Sep 7;5(9):e2229062. doi: 10.1001/jamanetworkopen.2022.29062 (PMC9453541; doi:10.1001/jamanetworkopen.2022.29062)
Supplement: Supplement. — eTable. Sample Demographics With and Without Multiple Imputation [file jamanetwopen-e2229062-s001.pdf]

Supplementary Online Content

Hill KA, Desai MM, Chaudhry SI, et al. Association of marginalized identities with Alpha Omega Alpha Honor Society and Gold Humanism Honor Society membership among medical students. *JAMA Netw Open*. 2022;5(8):e2229062. doi:10.1001/jamanetworkopen.2022.29062

eTable. Sample Demographics With and Without Multiple Imputation

This supplementary material has been provided by the authors to give readers additional information about their work.

© 2022 Hill KA et al. *JAMA Network Open.*

eTable . Sample demographics with and without multiple imputation

	Number (%)	
Characteristic	Sample without imputation (N = 33601)	Sample with imputation (N = 50384)
**Race**		
American Indian or Alaska Native	50 (0.2)	82 (0.2)
Asian	6675 (19.9)	10601 (21.0)
Black or African American	1597 (4.8)	2464 (4.9)
Hispanic, Latino, or of Spanish Origin	2247 (6.7)	3291 (6.5)
Multiracial	1687 (5.0)	2476 (4.9)
Native Hawaiian or Other Pacific Islander	16 (0.1)	26 (0.1)
White	20839 (62.0)	30610 (60.8)
Other	490 (1.5)	834 (1.7)
**Sex**		
Male	16822 (50.1)	25672 (51.0)
Female	16779 (49.9)	24712 (49.1)
**Sexual Orientation**		
Heterosexual	31519 (93.8)	47306 (93.9)
LGB	2082 (6.2)	3078 (6.1)
**Childhood Income**		
≥$75,000	21052 (62.7)	31748 (63.0)
$50,000-$74,999	5721 (17.0)	8160 (16.2)
$25,000-$49,999	4588 (13.7)	6864 (13.6)
<$25,000	2240 (6.7)	3611 (7.2)
**MCAT Score**		
<29	6993 (20.8)	10304 (20.5)
29-31	10529 (31.3)	15216 (30.2)
32-33	7074 (21.1)	10574 (21.0)
≥34	9005 (26.8)	14291 (28.4)

© 2022 Hill KA et al. *JAMA Network Open.*
